# What may cause fetus loss from acute pancreatitis in pregnancy: Analysis of 54 cases

**DOI:** 10.1097/MD.0000000000009755

**Published:** 2018-02-16

**Authors:** Min Tang, Jian-Ming Xu, Sha-Sha Song, Qiao Mei, Li-Jiu Zhang

**Affiliations:** a Department of Gastroenterology, the First Hospital of Anhui Medical University; b Department of Gastroenterology, the Second Hospital of Anhui Medical University, Hefei, China.

**Keywords:** acute pancreatitis in pregnancy, disease severity assessment, fetal loss, hyperlipidemia, non-stimulating experiment

## Abstract

Acute pancreatitis in pregnancy (APIP) poses a serious threat to the mother and her fetus, and might lead to fetal loss including miscarriage and stillbirth in certain patients. We sought to identify possible factors that affect fetal distress and evaluated outcomes of patients with APIP.

We retrospectively reviewed clinical records of 54 pregnant women with APIP, who were treated at 2 tertiary clinical centers over a 6-year period. Clinical characteristics including etiology and severity of APIP, fetal monitoring data, and maternofetal outcomes were analyzed.

Etiology of APIP included acute biliary pancreatitis (ABP, n = 14), hyperlipidemic pancreatitis (HLP, n = 22), and other etiologies (n = 18). Severity was classified as mild acute pancreatitis (MAP, n = 23), moderately severe acute pancreatitis (MSAP, n = 24), and severe acute pancreatitis (SAP, n = 7). The incidence of preterm delivery, fetal distress, and fetal loss increased with the progression of severity of APIP (*P* < .05). The severity of HLP was significantly higher than that of ABP and APIP of other etiology (*P* < .01). HLP was more likely to lead to fetal distress than other APs (*P* < .01). Only 12 (22.2%) patients had fetal monitoring including non-stress test (NST); 1 case of SAP (14.3%) and 15 cases of MSAP (62.5%) were not transferred to intensive care unit for intensive monitoring.

The incidence of fetal distress and fetal loss increased with worsening of APIP severity. HLP tends to result in worse fetal outcomes. The deficiencies of fetal state monitoring, lack of assessment, and management of pregnant women might increase the fetal loss in APIP.

## Abstract 2

Supplemental Digital Content is available in the text

## 1 Introduction

Acute pancreatitis in pregnancy (APIP) is a rare disease that typically presents as acute abdominal pain during pregnancy. Recently, the incidence of APIP has been reported to be as high as 1/1000.^[1,2]^ Acute progression of APIP may result in pancreatic necrosis, abscess, multiple organ dysfunction, and lead to other adverse maternofetal outcomes. Therefore, APIP greatly threatens maternal and fetal health. Due to advances in diagnostic and treatment technologies, maternal and fetal mortality rates have decreased significantly in recent years. One report^[3]^ in 2014 indicated a drop in APIP related maternal mortality from 37% to 0%, and a drop in APIP related fetal mortality from 60% to about 3%.

However, lack of physician's close attention is a major cause of fetal loss due to APIP. Sun et al^[4]^ first reported an overall fetal mortality of 23.2% in patients with APIP in 2011, and provided valuable data on fetal outcomes associated with APIP of different severity. Although the incidence of fetal death and stillbirth associated with severe pancreatitis was significantly higher than that associated with mild pancreatitis (5 vs 0 patients, *P* = .001), fetal loss rate was not significantly different between severe and mild APIP (*P* = .33). Further, the authors suggested that fetal loss associated with mild APIP was mainly due to miscarriage and abortion, whereas severe pancreatitis was more likely to lead to fetal death and stillbirth. However, a detailed analysis of APIP related fetal loss has not been reported in recent years.^[5–8]^ A recent case report^[9]^ described a case of mild APIP, who had responsive non-stress test (NST) and normal biophysical profile score (BPS) at admission. However, this patient experienced sudden deterioration in clinical condition accompanied with increase in biochemical markers after admission, and ended up with an emergency cesarean section on the third day of admission. A 2300 g stillborn male fetus was delivered with conspicuous generalized peeling skin lesions. This case inspired further thought as to why a pregnancy with mild pancreatitis ended up with intrauterine fetal death. Are there any deficiencies in fetal monitoring and evaluation, and management of APIP patient?

What may cause fetus loss from acute pancreatitis in pregnancy?

To answer the questions above, we retrospectively collected and analyzed clinical data of patients with APIP treated at 2 tertiary centers from 2009 to 2015. The objective of the present study was to identify factors that might affect fetal outcomes in patients with APIP, and to examine the necessity of enhanced evaluation of severity of APIP and close fetal monitoring in these patients.

## 2 Materials and methods

The present study was approved by the Ethics Committee at the Anhui Medical University. We retrospectively analyzed clinical data of patients with APIP from 2 tertiary care centers (1st and 2nd Affiliated Hospitals of Anhui Medical University), over a 6-year period from 2009 to 2015. Out of 3784 medical records of pregnant women at the 2 tertiary centers, 54 cases of APIP were included in this study.

### 2.1 Diagnostic and classification criteria of the severity of AP

According to the guidelines for the diagnosis and treatment of AP in China,^[10]^ the diagnosis of acute pancreatitis requires 2 out of the 3 criteria: abdominal pain consistent with acute pancreatitis (persistent severe epigastric pain, acute onset, typically radiates to the back); serum lipase activity (or amylase activity) at least 3 times greater than the upper limit of the normal reference range; and characteristic findings of acute pancreatitis on contrast-enhanced computed tomography (CECT), or, less commonly, on magnetic resonance imaging (MRI) or transabdominal ultrasonography. Based on the presence of persistent organ failure and local or systemic complications, the severity of AP is classified into MAP, MSAP, and SAP.

### 2.2 Criteria for patient selection

Women who were diagnosed as AP during pregnancy or within 1 week postpartum were included in this study. The exclusion criteria were: non-pregnant women; onset of AP occurred beyond 1-week postpartum; presence of other severe disease such as acute fatty liver of pregnancy, gestational hypertension, preeclampsia and gestational diabetes.

### 2.3 Etiologies of APIP

The most common etiology of APIP is gallstones, followed by alcohol abuse, and hyperlipidemia.

According to the Chinese guidelines for acute pancreatitis^[10]^ and study of hyperlipidemic pancreatitis (HLP),^[11]^ AP patients were diagnosed as hypertriglyceridemia-induced acute pancreatitis (HTGP) if they qualified the following criteria: serum triglyceride (TG) > 11.3 mmol /L, or between 5.65 and 11.3 mmol/L (500–1000 mg/mL) but with a lipemic serum, and in the absence of biliary disease, alcohol, or medication abuse. Acute biliary pancreatitis (ABP) was defined as AP associated with gallstones or sludge in the biliary tree or the gallbladder. Pancreatitis in patients with history of alcohol abuse was classified as alcoholic pancreatitis. Pancreatitis associated with high-fat diet, acute exacerbation of chronic pancreatitis, infectious pancreatitis, pancreatitis caused by sphincter of oddi dysfunction (SOD), and pancreatitis of idiopathic etiology was classified under the category “other etiology.” Pancreatitis caused by gallstones in patients with hyperlipidemia was also regarded as HLP.

### 2.4 Maternal and fetal outcomes

Different trimesters of pregnancy were defined as: first (1–12 weeks); second (13–28 weeks); and third (≥29 weeks) trimester. Term pregnancy was defined as ≥37 completed weeks of gestation, and included spontaneous term labor, termination of pregnancy at term by cesarean section because of maternal or fetal complications, and induction of labor with vaginal delivery. Preterm pregnancy (28–36 weeks of gestation) included both delivery by cesarean section because of maternal or fetal indications and spontaneous preterm labor with vaginal delivery. Abortion <28 weeks of gestation included both spontaneous and medically-induced abortion.

Intrauterine fetal distress was defined as fetal movements <10 times within a 12-hour period on self-monitoring; baseline of fetal heart beat >160 or <120 beats per minute; non-reactive NST, or BPS ≤5. NST and BPS are important means to evaluate fetal distress. NST was performed and evaluated based on the variations of fetal heart beat under non-contraction stimulation. BPS is assessed on electronic fetal heart rate monitoring with ultrasound and cardiotocography. Five parameters including, NST, fetal respiratory movement, gross body movement, fetal tone, and amniotic fluid volume were evaluated in BPS, and making a total score of 10. BPS no >5 indicated fetal distress. Fetal loss included abortion, fetal demise, and stillbirth. Death occurring prior to 28 weeks of gestation was classified as an abortion, and death occurring after 28 weeks was classified as fetal demise or stillbirth.

### 2.5 Assessment of APIP

We collected the following clinical data from pregnant women with AP, Ronson evaluation on admission, acute physiology and chronic health evaluation (APACHE) II score, fetal monitoring (including NST, BPS, transabdominal ultrasonography). The severity of AP were re-evaluated based on the Chinese guidelines for diagnosis and treatment of AP^[10]^ and the modified Marshall scoring system for AP.^[12]^ Patients who received medical care from both obstetricians and intensive care unit (ICU) specialists or general surgeons were considered to have received medical care from a multidisciplinary team (MDT).

### 2.6 Statistical analysis

Data analysis was performed using SPSS 17.0 software (SPSS Inc. Chicago, IL). Categorical variables are presented as frequencies and percentages (n [%]); continuous variables are presented as median (range). Between-group differences were assessed by Chi-squared test, Mann–Whitney *U* test or Fisher exact test, as applicable. *P* < .05 was considered as statistically significant. The etiology of AP was classified as HLP and non-HLP; the severity of AP was classified as MAP, MSAP, and SAP.

## 3 Results

### 3.1 Basic clinical characteristics

Over the study period, 54 cases (37 from the 1st Hospital and 17 from the 2nd Hospital) of APIP were identified among the 39,416 deliveries, which corresponded to an incidence of 1.37 cases of APIP per 1000 pregnant women. Mean age of patients with APIP was 27.26 years old (range, 19–39). Fifteen (27.8%) of study population were multiparous and 39 (72.2%) were primiparae. There were 53 singleton pregnancies and 1 twin pregnancy which lasted until 25 weeks with acute onset of APIP and intrauterine fetal death. The incidence of APIP during the first trimester was 3.7% (2 patients), 33.3% (18 patients) during the second trimester, 59.3% (32 patients) during the third trimester, and 3.7% (2 cases) occurred at postpartum period. The average gestation age at presentation was 29.1 ± 7.9 weeks. Detailed clinical characteristics of patients transferred to ICU are listed in Supplemental Table 1.

### 3.2 Etiologies and severities of APIP

The detailed etiologies and severities of all patients at different trimesters are listed in Table 1. Among 54 patients, 14 (25.9%) were identified as ABP, and 40.7% (22 cases) were HLP (including 2 patients) with gestational diabetes, and 1 patient diagnosed with cholelithiasis detected on ultrasonography, but who had normal liver function (alanine aminotransferase [ALT] 20 U; aspartate aminotransferase [AST] 20 U; gamma-glutamyl transferase [GGT] 30 U; alkaline phosphatase [ALP] 89 U; total bilirubin [TBIL] 24 mmol/L; total bile acid [TBA] 2.0 mmol/L; triglyerides [TG] 16.62 mmol/L; total cholesterol [TC] 29.11 mmol/L). The remaining 18 patients (33.3%) included 2 patients with acute exacerbation of chronic pancreatitis, 2 patients with infectious pancreatitis, 2 patients with suspected sphincter of oddi dysfunction (SOD), 9 patients with pancreatitis of idiopathic etiology, and 3 patients having pancreatitis because of high-fat diet.

**Table 1:**
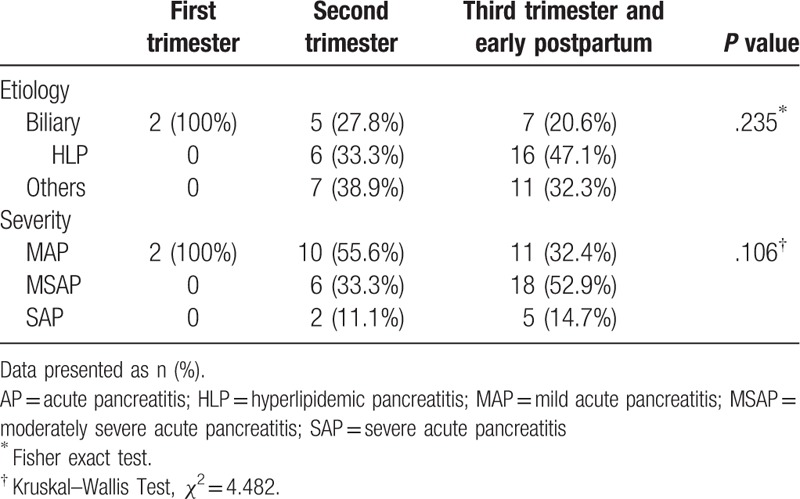
Etiology and severity of APIP in different trimesters.

Patients were categorized as MAP (n = 23), MSAP (n = 24), and SAP (n = 7). There were no significant differences with respect to various etiologies of AP occurring at different trimesters (Table 1). However, HLP tended to increase with the progression of gestational age (0 vs 33.3% vs 47.1% in 1st, 2^nd^, and 3rd trimester, respectively; Table 1). Likewise, occurrence of MSAP and SAP tended to increase with the progression of gestational age (MSAP: 0 vs 33.3% vs 52.9%, respectively; SAP: 0 vs 11.1% vs 14.7%, respectively; Table 1).

The severities of APIP in different etiologies are clearly listed in Table 2. Significant between-group differences were observed in the severity of AP disaggregated by etiologies (Table 2, Kruskal–Wallis test, Chi-square = 25.476, *P* < .01). Severity of AP in patients with HLP was significantly worse than that in ABP (Mann–Whitney *U* test, hyperlipidemia vs biliary *z* = –3.912, *P* < .01).

**Table 2:**
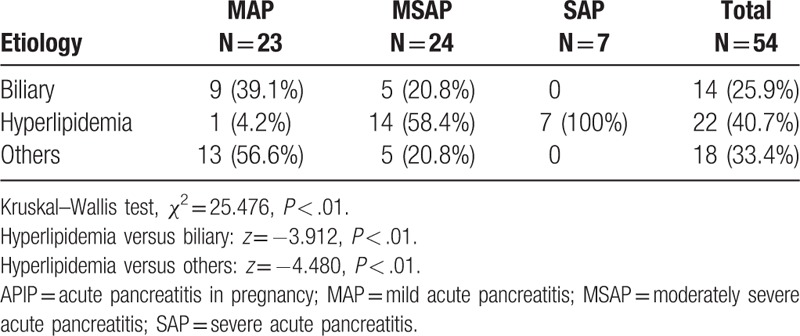
Severity of APIP disaggregated by underlying etiologies.

### 3.3 Fetal distress and fetal loss based on APIP etiologies

The incidences of fetal distress and fetal loss disaggregated by etiologies of APIP are presented in Table 3. The present data suggest that hyperlipidemia might have a stronger association with fetal distress as compared with other etiologies (*χ*^2^ = 11.477, *P* < .01). There was no significant difference in the incidence of fetal loss associated with APIP of different etiologies (*P* = .203).

**Table 3:**
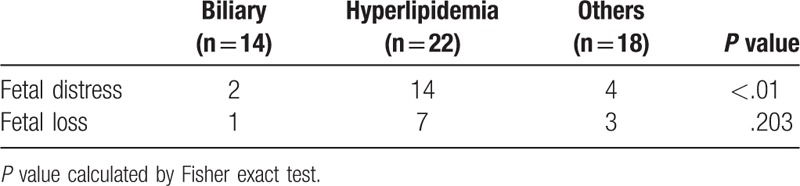
Fetal distress and fetal loss based on APIP etiologies.

### 3.4 Fetal monitoring

Prosecutions of fetal monitoring in different severities of APIP are listed in Table 4. Although all patients underwent routine ultrasound, only 36 patients underwent fetal ultrasound examination and only 12 of them underwent NST (22.2%). In particular, only 1 out of the 7 patients with SAP received NST monitoring, while 75% of MSAP patients did not receive NST monitoring.

**Table 4:**
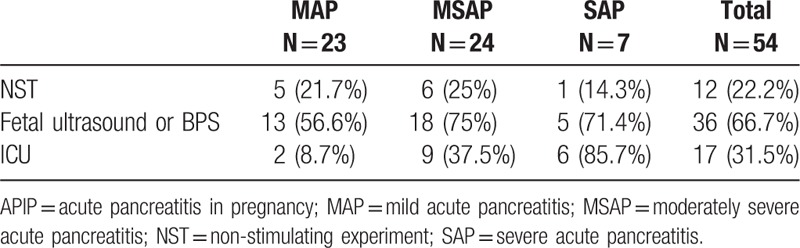
Maternal and fetal monitoring.

For patients who received MDT management, percutaneous peritoneal drainage was performed for 2 patients with MSAP and 1 patient with SAP for symptomatic relief. A stent was placed by ERCP for 1 patient with bile duct dilatation caused by SOD. Seventeen patients were transferred to ICU, but still there was 1 case of SAP (14.3%) and 15 cases of MSAP (62.5%) that were not transferred to ICU for intensive monitoring. Detailed fetal monitoring and management, as well as maternal and fetal outcomes are listed in Supplemental Tables 1 and 2.

### 3.5 Relationship between severities of APIP and maternal and fetal outcomes

Detailed maternal and fetal outcomes are listed in Table 5 and Supplemental Table 2. No maternal deaths occurred in this study population. Fetal loss occurred in 11 patients (20.4%); these included fetal demise in 8 patients (14.8%). A total of 31 (57.4%) pregnant women delivered at term pregnancies, while pregnancy was actively terminated in 5 women (3 cesarean sections with fetal survival and 2 cases of fetal death). Twelve women (22.2%) had preterm termination of pregnancy which included 8 cesarean sections; fetus survived in 9 cases while fetal death occurred in 3 cases. There were 6 cases of abortion and fetal loss, 3 cases of fetal death, while 1 woman who had twin pregnancy experienced spontaneous abortion. Maternal and fetal outcomes disaggregated by severity of AP are summarized in Table 5. We found that MAP was more likely to result in spontaneous term delivery as compared with moderately severe and severe AP (*P* < .001). The incidence of preterm delivery, fetal distress, and fetal loss increased with the progression of APIP severity (*P* < .05).

**Table 5:**
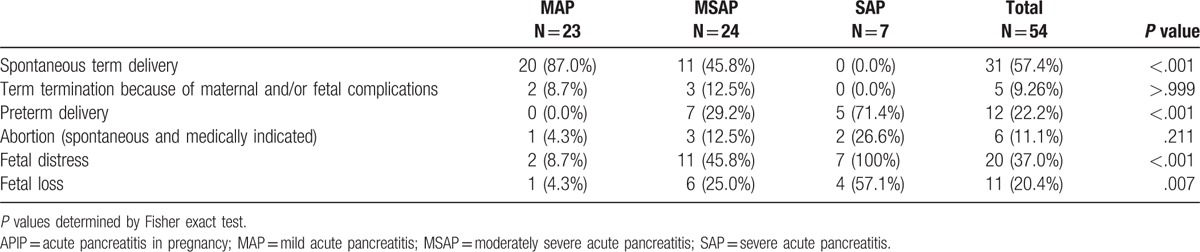
Maternal and fetal outcomes of patients with APIP.

## 4 Discussion

Since APIP poses a serious threat to the life of both mother and fetus, it is essential to define the onset of APIP. Although there is no defined standard to determine the onset of APIP, several studies^[13]^ have suggested that AP that occurs soon after parturition should also be included as APIP. According to our experience, misdiagnosis and missed diagnosis tend to be quite common among APIP patients. Some of these patients might have had APIP during pregnancy but were only diagnosed in the early postpartum period. Therefore, patients who were diagnosed with AP during early postpartum period were also included in this study.

ABP is the most common etiology of AP among pregnant women in Europe and America (65–100%); HLP and alcoholic pancreatitis account for about 5% to 15%, and 5% to 12% of cases. Other causes include idiopathic pancreatitis, drug-induced pancreatitis, traumatic pancreatitis, pregnancy-induced hypertension, acute fatty liver of pregnancy, and genetic disorders.^[1,3,14]^In China, few pregnant women consume alcohol during pregnancy, but most of them tend to have high-fat diet due to local culture. Besides, the serum lipid levels of gravida are liable to be affected by estrogen, progesterone, human chorionic gonadotropin (HCG), and other hormones. Plasma triglyceride (TG) levels can rise 2 to 4 folds during pregnancy.^[15]^ As expected, the incidence of HLP was much higher than ABP (40.7% vs 25.9%) among Chinese women in the present study. The same situation was also reported in Korean. Among 5 cases of APIP reported in South Korea in 2005, 4 of them was because of hyperlipidemia and only 1 case of alcohol-induced APIP.^[16]^ Therefore, the etiology pattern of APIP differed significantly between Asian and European women. So far, the pathophysiological mechanisms of HLP are still not clear, especially its influence on placenta function. Some researchers have proposed that in severe HTG, pancreatic lipase diffusing from pancreatic acinar cells to the interstitial cell side hydrolyses TGs into chylomicrons (CMs) and very low density lipoproteins. This would generate free fatty acids (FFAs), which in turn induce damage to the vascular endometrial cells and pancreatic acinar cells, as well mitochondrial toxicity, and thus pancreatic cell injury.^[17–20]^ In a recent study, some fatty acids were shown to inhibit mitochondrial complexes I and V and induced mitochondrial injury and necrosis.^[19,21]^ Acute pancreatitis can also be complicated with other severe conditions, for instance, pancreatic necrosis, severe slectrolyte disorders, preecampsia, and even acute respiratory distress syndrome.^[14,21]^ HLP, therefore, is more likely to be associated with significant mortality for mother and fetus.^[14]^ On the other hand, lipoprotein synthesis is closely related with increased estrogen levels and insulin resistance, the triglyceride levels could elevated to 2 to 3 times higher than non-pregnant levels. Particularly, the insulin resistance is more obvious during the 3rd trimester, which results in more significant hypertriglyceridemia as well as the incidence of APIP.^[22]^ In agreement with above reports, we found that pregnant women with HLP were more likely to have fetal intrauterine distress (14/22, 63.6%) in the present study.

Damage to placenta during APIP has been rarely reported. Cheang et al^[23]^ in 2007 reported 1 case of acute necrotic pancreatitis complicated with uteroplacental apoplexy, which had systemic inflammatory response syndrome (SIRS) for more than 48 hours. Researchers postulated that the occurrence of uteroplacental apoplexy might be due to SIRS, which would cause massive damage to almost all systems, including respiratory system, digestive system, cardiac function, renal function, immune function,^[24]^ and possibly placenta in this particular case. A few studies in animal models have documented pancreatitis-related placenta injury. In 1 study,^[25]^ expression of E-selectin in serum and placenta tissues markedly increased 1 hour after induction of pancreatitis in an APIP rat model. The concentration of E-selectin was significantly related to the degree of pancreatic and placenta injury. Based on a thorough investigation of pancreatitis related plancental injury, researchers suggested that placental injury during pancreatitis might be related with activation of mitogen activated protein kinases pathway, particularly, c-Jun N-terminal kinase and p-38.^[26]^ However, the detailed mechanisms of fetal loss caused by AP-induced placental damage require further investigation.

In this study, the severity of APIP was closely associated with maternal and fetal outcomes. Our study provides more detailed and more definitive evidence than that reported by Sun et al.^[4]^ No maternal deaths occurred in our study; however, 11 (20.4%) cases of fetal loss occurred, 8 (14.8%) of whom were stillbirths. The incidence of preterm delivery, fetal distress, and fetal loss also increased with the progression in severity of APIP. Fetal loss was much higher in MSAP and SAP groups as compared with that in the MAP group. These findings highlight the significance of assessment for APIP on admission, prognostic evaluation of patients with MSAP and SAP, and careful fetal monitoring.

NST and BPS are the most commonly used modalities for assessment of fetal well-being. In this study population, only 36 patients received fetal ultrasound evaluation and 12 (22.2%) of them received NST (6 [25%] patients in the MSAP group and only 1 [14.3%] patient in the SAP group). This scenario might be due to insufficient attention paid to pregnant women with APIP and insufficient fetal monitoring. In a randomized clinical trial,^[27]^ acoustic stimulation test (AST) and feeding mother stimulation, helped to reduce the false positive rate and increased negative predictive value of non-reactive NST. A combination of NST and stimulation response thus seems to be a convenient, fast and safe approach to evaluation of fetal well being. The relative complexity and time-consuming nature of BPS limits its wider clinical application. Other indicators including systolic–diastolic ratio (S/D) by fetal ultrasound examination could also serve as valuable indicators of intrauterine fetal distress and poor prognosis of fetuses.^[28]^ Therefore, detection of S/D may be a much more convenient method for use in clinical settings. One meta analysis^[29]^ suggested that with the use of Doppler ultrasound in women with high-risk pregnancy appeared to improve a number of obstetric care outcomes and to reduce perinatal deaths. Therefore, we suggest NST combined with S/D as the first-line monitoring method. BPS and other tests could be provided, if necessary.

A team of MDT comprising of a gastroenterologist, an ICU specialist, an obstetrician, and a general surgeon is highly recommend to minimize the incidence of fetal loss. One case of SAP (14.3%) and 15 cases of MSAP (62.5%) were not transferred to ICU for intensive monitoring. This lack of MDT management of pregnant women with APIP could be 1 possible reason of fetal loss. All clinicians in the MDT team should be proficient in the assessment of AP and fetal monitoring. Some patients might have non-specific abdominal pain, which could easily be misdiagnosed as other obstetrical problems. The involvement of gastroenterologist, obstetrician, surgeon, and ICU specialist could help in the differential diagnosis of APIP and other problems with similar symptomalogy such as gestational diabetes, pregnancy-induced hypertension, eclampsia, hemolysis, elevated liver enzymes and low platelet (HELLP) syndrome, fatty liver of pregnancy syndrome, appendicitis, and cholecystitis. The health care from MDT team can provide sufficient and in-depth care for each patient, and therefore improve the fetal outcomes.

More careful fetal and maternal monitoring and assessment on admission is required. Progression of maternal and fetal well-being should be informed promptly to every clinician in the MDT team for a more detailed and through evaluation of fetal and maternal conditions. Clinicians need to avoid iatrogenic injury, including use of medicines (especially fenofibrate) and x-ray exposure to the fetus. MRI was also reported to potentially induce overheating of tissues and might interfere with fetal development,^[30]^ and hence should be avoided in the first trimester.

The management of APIP should be individualized based on gestational age^[3]^ Conservative management of APIP was suggested for patients in first trimester, and laparoscopy can be provided for patients in the second trimester. For patients in the third trimester, either conservative management or ERCP until delivery, or laparoscopy in early postpartum period.^[3]^ Some researchers^[31]^ reported that ERCP which involves no radiation exposure is safe and effective for the treatment of choledocholithiasis during pregnancy. Some recent reports^[32]^ suggested a combination of intravenous heparin and insulin infusion in severe cases of gestational hypertriglyceridemia-induced AP, which increased the lipoprotein lipase activity. Besides, even though no definitive clinical guidance is available, use of plasmapheresis and hemofiltration have also been reported to be helpful in some cases.^[5]^

Diligent decision-making related to the termination of pregnancy in patients with APIP, and its timing are important to minimize fetal loss. Termination of pregnancy should be considered in case of clinical deterioration despite 24 to 48 hours of active treatment of moderate and severe cases, paralytic ileus, stillbirth, fetal malformation, severe pancreatitis, and severe fetal distress.^[33]^

This study still has several limitations that should be mentioned. First of all, it is a retrospective study recruited clinical data of women had APIP. The retrospective nature of the present study will limit the application of study results. However, a prospective study to compare APIP women receive either extensive or no MDT care, as well as careful fetal monitoring or no monitoring, cannot be possible due to ethic concerns. Secondly, a small study population might be another drawback of this study. Because of the low incidence of APIP, to recruit a large number of study population is a quite difficult task. This study had collected data from 2 tertiary center during 6 years, >50 cases of APIP had been the largest study population as far as we know. Moreover, because of rarity of APIP, there is no guideline available to provide a standard management of APIP. Chinese guideline of acute pancreatitis was used to identify patients with APIP, which might limit the worldwide application of the study results. Fortunately, Chinese guideline was established based on American College of Gastroenterology Guideline: management of acute pancreatitis and International Association of Pancreatology treatment guideline of acute pancreatitis,^[34,35]^ and thus the study results can also shed light on the management of other study populations. Besides, since this study conducted at 2 tertiary centers, patients with more severe conditions were transferred to out centers. The incidence of APIP could be much higher than in general population.

In conclusion, APIP poses a serious threat to the safety of the mother and fetus. The incidence of fetal distress and fetal loss increased with the progression of severity of APIP. HLP in pregnancy is associated with more intrauterine fetal distress as compared with APIP by other etiologies. The deficiencies of fetal state monitoring, lack of assessment, and management of pregnant women might be the main cause of increased fetal loss from APIP. More careful assessment and monitoring of maternal and fetal conditions, as well as enhanced management from MDT, are essential to improve maternal and fetal outcomes.

## Supplementary Material

Supplemental Digital Content
