# Supplementary material for: What may cause fetus loss from acute pancreatitis in pregnancy: Analysis of 54 cases
Source: Medicine (Baltimore). 2018 Feb 16;97(7):e9755. doi: 10.1097/MD.0000000000009755 (PMC5839860; doi:10.1097/MD.0000000000009755)
Supplement: Supplemental Digital Content [file medi-97-e9755-s001.docx]

Supplemental **Table 1: Clinical data of 17 patients referred to the intensive care unit**

| **Case number** | **ICU days** | Duration of hospital stay | **Severity of APIP** | **Maternal outcome** | **Fetal outcome** | Apgar score | **Birth weight** |
| --- | --- | --- | --- | --- | --- | --- | --- |
| **3** | **6** | **21** | **MSAP** | **Preterm delivery by cesarean section** | **survival** | **7** | **1980 g** |
| **4** | **4** | **14** | **MSAP** | **Preterm delivery medically indicated** | **survival** | **7** | **2500 g** |
| **9** | **4** | **11** | **MSAP** | Spontaneous term delivery | **survival** | **--** | **--** |
| **15** | **4** | **16** | **SAP** | **Preterm vaginal delivery** | **survival** | **8** | **--** |
| **18** | **6** | **33** | **SAP** | **induced abortion** | **fetal death** | **--** | **--** |
| **19** | **6** | **21** | **SAP** | **Preterm delivery medically indicated** | **fetal death** | **--** | **--** |
| **20** | **2** | **12** | **MSAP** | **Preterm delivery by cesarean section** | **survival** | **10** | **2335 g** |
| **23** | **9** | **21** | **SAP** | **Preterm delivery by cesarean section** | **survival** | **4** | **2150 g** |
| **28** | **17** | **30** | **MSAP** | **Medically indicated**  **Term termination** | **fetal death** | **0** | **2580 g** |
| **29** | **12** | **32** | **MSAP** | **Medically indicated**  **preterm delivery** | **fetal death** | **0** | **3150 g** |
| **36** | **7** | **24** | **SAP** | **induced abortion** | **fetal death** | **--** | **--** |
| **40** | **1** | **13** | **MSAP** | **Preterm delivery by cesarean sections** | **survival** | **8** | **1750 g** |
| **43** | **10** | **21** | **SAP** | **Preterm delivery by cesarean section** | **fetal death** | **0** | **3500 g** |
| **44** | **2** | **11** | **MAP** | **Term termination by cesarean section** | **survival** | **10** | **--** |
| **46** | **4** | **25** | **MSAP** | Spontaneous term delivery | **Survival** | **--** | **--** |
| **50** | **1** | **11** | **MSAP** | Spontaneous **abortion** | **twins fetal death** | **0/0** | **--** |
| **53** | **2** | **9** | **MSP** | **Term termination**  **cesarean section** | **Survival** | **1** | **2880 g** |

IP, Acute pancreatitis in pregnancy; MAP, mild acute pancreatitis; MSAP, moderately severe acute pancreatitis; SAP, severe acute pancreatitis; ICU, intensive care unit

**Supplemental Table 2: Clinical outcomes of 12 patients with NST non-stimulating experiment**

| **Case number** | **NST outcome** | **Severity of APIP** | **Maternal outcome** | **Fetal outcome** | Apgar score | **Birth weight** |
| --- | --- | --- | --- | --- | --- | --- |
| **8** | **No reaction** | **MSAP** | **Medically indicated**  **termination at term** | **fetal death** | **0** | **--** |
| **29** | **6 points**  **no reaction** | **MSAP** | **Medically indicated**  **preterm delivery** | **fetal death** | **0** | **3150 g** |
| **33** | **Fast fetal heart rate** | **MSAP** | **Induced abortion** | **Fetal loss** | **--** | **--** |
| **40** | **Unspecified** | **MSAP** | **Preterm delivery by cesarean sections** | **survival** | **8** | **1750 g** |
| **41** | **Unspecified** | **SAP** | **Preterm delivery by cesarean sections** | **survival** | **6** | **2060 g** |
| **42** | **8 points**  **Reaction positive** | **MSAP** | **Termination at term by cesarean section** | **survival** | **9** | **3100 g** |
| **44** | **Unspecified** | **MAP** | **Term termination by**  **cesarean sections** | **survival** | **10** | **--** |
| **48** | **Unspecified** | **MAP** | Spontaneous term delivery | **survival** | **10** | **3100 g** |
| **49** | **Unspecified** | **MAP** | Spontaneous term delivery | **survival** | **10** | **3100 g** |
| **51** | **Unspecified** | **MAP** | Spontaneous term delivery | **survival** | **10** | **3750 g** |
| **52** | **Unspecified** | **MSAP** | Spontaneous term delivery | **survival** | **--** | **--** |
| **53** | **Doubtful**  **no reaction** | **MSP** | **Term termination**  **cesarean section** | **survival** | **10** | **2880 g** |

IP, Acute pancreatitis in pregnancy; MAP, mild acute pancreatitis; MSAP, moderately severe acute pancreatitis; SAP, severe acute pancreatitis; NST, non stimulating experiment
